# Examining the pace of change in contraceptive practices in abortion services – a follow-up case study of a quality improvement collaborative

**DOI:** 10.1186/s12913-020-05799-x

**Published:** 2020-10-16

**Authors:** Helena Kilander, Jan Brynhildsen, Siw Alehagen, Johan Thor

**Affiliations:** 1 Department of Obstetrics and Gynaecology, Eksjö Hospital, Region Jönköping County, Sweden; 2 Department of Health, Medicine and Caring Sciences , Linköping University, Linköping, Sweden; 3 Department of Obstetrics and Gynaecology and Department of Biomedical and Clinical Sciences, Linköping University, Linköping, Sweden; 4 Department of Obstetrics and Gynecology, Faculty of Medicine and Health, Örebro University, Linköping, Sweden; 5 Jönköping Academy for Improvement of Health and Welfare, School of Health and Welfare, Jönköping University, Jönköping, Sweden

**Keywords:** Health services accessibility, Long- acting reversible contraception, Contraceptive counselling, System performance, Quality improvement, Professional development and pregnancy termination

## Abstract

**Background:**

Among all women who experienced an abortion in Sweden 2017, 45% had previously underwent at least one abortion. This phenomenon of increasing rates of repeat abortions stimulated efforts to improve contraceptive services through a Quality Improvement Collaborative (QIC) with user involvement. The participating teams had difficulty in coordinating access post-abortion to the most effective contraception, Long-acting reversible contraception (LARC), during the eight-month QIC. This prompted questions about the pace of change in contraceptive services post-abortion. The aim of the study is to evaluate the evolution and impact of QIC changes regarding patient outcomes, system performance and professional development over 12 months after a QIC designed to enhance contraceptive services in the context of abortion.

**Methods:**

This follow-up case study involves three multi-professional teams from abortion services at three hospitals in Sweden, which participated in a QIC during 2017. We integrated qualitative data on the evolution of changes and quantitative data regarding the monthly proportion of women initiating LARC, analysed in statistical control charts from before the QIC up until 12 months after its conclusion.

**Results:**

Teams A and B increased the average proportion of women who initiated LARC within 30 days post abortion in the 12 months after the QIC; Team A 16–25%; Team B 20–34%. Team C achieved more than 50% in individual months but not consistently in the Post-QIC period. Elusive during the QIC, they now could offer timely appointments for women to initiate LARC more frequently. Team members reported continued focus on how to create trustful relationships when counseling women. They described improved teamwork, leadership support and impact on organizing appointments for initiating LARC following the QIC.

**Conclusions:**

QIC teams further improved women’s timely access to LARC post abortion through continued changes in services 12 months after the QIC, demonstrating that the 8-month QIC was too short for all changes to materialize. Teams simultaneously improved women’s reproductive health, health services, and professional development.

## Background

The context of an abortion represents an opportunity to help women to prevent repeat unintended pregnancies (UPs) [1, 2]. Access to effective contraceptive methods post-abortion, Long-Acting Reversible Contraception (LARC) in particular, is essential for women’s sexual and reproductive health [2, 3]. Moreover, women who use LARC in general report higher rates of satisfaction and 12-month continuation than users of other contraceptive methods [4]. However, the proportion of women who experience repeat abortions in Sweden, as in the UK, is increasing [5, 6], despite growing awareness of women’s needs for respectful guidance in contraceptive counselling [7–9] and timely access to LARC [7, 10].

We recently reported on a Quality Improvement Collaborative (QIC) with user involvement, where three participating teams tested evidence-based changes to improve contraceptive services at the time of abortion [11]. Participating healthcare professionals (HCPs) developed a more person-centred approach to contraceptive counselling in the context of an abortion. None of the teams, however, reached their shared goal of providing ≥50% of women with access to LARC within 30 days post-abortion [11]. The QIC’s 8 month duration may have been too short to successfully implement all intended changes, a common challenge in complex health care systems [12].

Batalden and Davidoff define health care quality improvement as the combined efforts of all stakeholders, including patients, that yield changes in several dimensions [12]: “patient outcomes (better health), system performance (better care), and professional development (learning, joy in work) [13]”. There is growing support for the benefits of the QIC approach and of user involvement when improving health care services [13–15]. A study of twelve family planning teams in the US indicated that a QIC stimulated the improvement of contraceptive services [15]. The long-term fate and impact of QICs in health care systems, however, especially regarding contraceptive services in abortion care, are still poorly understood.

## Methods

Drawing on Batalden and Davidoff’s definition, [13] this study aimed to evaluate the evolution and impact of QIC changes in regard to patient outcomes, system performance, and professional development over 12 months after a QIC designed to enhance contraceptive services in the context of abortion [11]. This report draws on the SQUIRE guidelines (www.squire-statement.org) [16].

### Setting and improvement approach

The QIC brought together teams from abortion services at three departments of obstetrics and gynaecology in south-eastern Sweden; two in mid-sized county-level hospitals (teams A and B) and one in a smaller district hospital (team C). The teams volunteered to join the QIC as part of fulfilling the requirements for health services to undertake quality control and improvement. The QIC drew on the Breakthrough Collaborative model and promoted use of Plan-Do-Study-Act cycles [17]. An interdisciplinary researcher group and an improvement advisor developed, supported, and studied the QIC. It involved four learning sessions (LS) from March to November 2017, with action periods in between when teams tested changes in their practice settings. As a form of user involvement, two women with lived experience of contraceptive counselling at the time of an abortion shared their (de-identified) user perspectives on the teams’ proposed changes in order to help the teams to improve the counselling and services [11].

Using a Driver diagram [18], the teams were introduced to four primary drivers for improvement at the time of an abortion, based on previous research evidence [7, 19, 20]:

- Providing information about contraceptive methods in a better way before, during, and after the first visit.
- Developing a respectful approach to counselling, including on contraception, at the time of an abortion.
- Improving counselling and services to women with communication needs.
- Developing better access to LARC at the time of an abortion.

Developing a respectful approach to counselling included for example, to actively include women in the conversation and the decision about subsequent contraception, as well as to use more open questions about contraception, in line with person-centered counselling. These changes were intended to better meet women’s individual needs, to facilitate their choice of contraception, and to prevent HCPs from “pushing” skeptical women to choose LARC against their will.

Women with communication needs, defined here as women with language barriers (i.e. who do not speak Swedish), women with mental health problems, women who have experienced repeat abortions and women who decline offers of contraception, seem to have special needs for individually tailored counselling [7].

### Study design

This study is a follow-up of the initial case study [11], combining qualitative and quantitative data collected at six and 12 months after the concluding QIC LS in November 2017. Data concern the fate and impact of the teams’ QIC changes.

### Data collection

Qualitative data consisted of *field notes* from follow-up QIC team meetings at six and 12 months post-QIC, as well as the first author’s e-mail correspondence, and notes from telephone conversations with team members.

Quantitative data covered the number of induced abortions and the number of women per month at each site who received LARC within 30 days after the abortion. Data from abortion associated with foetal abnormalities were excluded. The ‘time of an abortion’ was defined as the time of mifepristone intake (medical abortion) or the day of surgical abortion. Data managers from participating regional health systems extracted, de-identified, and reported data (aggregated per month to protect patient privacy) from administrative information systems regarding the number of abortions and the number of LARC insertions within 30 days post-abortion within the catchment area of each hospital. These data formed the monthly proportion of women who received LARC within 30 days.

### Data analysis

We performed qualitative content analysis of the data according to the framework of Patton [21] from field notes and project documentation. Author HK read through all the data, and coded and arranged the data in categories matching the four primary drivers developed within the QIC [11]. We then analysed the qualitative data deductively based on Batalden and Davidoff’s framework [13] regarding patient outcomes, system performance, and professional development. Three other researchers (JT, JB and SA) reviewed and refined the analysis individually. The authors discussed and agreed on the analysis through consensus.

As in the initial QIC [11], quantitative data were analysed chronologically using Statistical Process Control charts to identify signs of statistically significant change in performance over time, identified as special cause (non-random) variation [22]. We reviewed the monthly proportion of women who had undergone an abortion and who received LARC within 30 days after the abortion.

We applied three rules to identify statistically significant change:

- A run of six to eight or more points on one side of the center line.

-Two out of three consecutive points appearing beyond 2 SD on the same side of the center line (ie, two-thirds of the way towards the control limits).

- A run of six or eight (some prefer seven) or more points all trending up or down. (Mohammed et al. 2008), [23].

## Results

All three teams participated in two follow-up webinar meetings, at 6 months and 12 months post-QIC. At 6 months, 12 team members and three researchers participated, and at 12 months, five team members and one researcher (HK) took part. Over these 12 months, two of the teams experienced a high turnover of midwives.

The teams reported that during the 12 months after the QIC, they had maintained QIC changes in clinical practice regarding contraceptive counselling and that women’s access to LARC had improved. Furthermore, they reported how they had tested additional evidence-based improvement actions, guided by the driver diagram, which they had not tested during the QIC (Table 1).

**Table 1:** Summary of changes and improvements in contraceptive counselling and services 12 months after the QIC

The four driver categories	Changes in clinical practice *Subcategories*	Team A		Team B		Team C	
		Maintain	New test	Maintain	New Test	Maintain	New Test
**Provide information about** **contraceptives in a better way before, at and after the first visit.**	- Prepare women better, when women call to make an appointment for abortion counseling, that the visit will include contraceptive counselling	X			N	X	
	- Use a visual tool for information about the effectiveness of different contraceptives	X		X		X	
	-Use and hand out an information leaflet about side effects and effectiveness of different contraceptives when women choose contraceptives					X	
	-Use and hand out an information leaflet about side effects when women choose contraceptives	x		x			
	-Go over both positive and negative side effects regarding contraceptives	X		X		X	
	-Show prototypes for different contraceptive methods		N		N	X	
**Develop a respectful approach to counseling, including on contraception, at the time of an abortion**	-Introduce and use “Do not disturb” signs					X	
	-Schedule more flexible time for contraceptive counselling					X	
	-Avoid scheduling less interested physicians when making abortion counselling appointments					X	
	-Use more open questions in the conversation about contraception	x		x		x	
	- Actively include women in the conversation and decision about contraceptives	x		x		x	
**Develop better access to LARC at the time of an abortion**	-Add more appointments for IUD-insertion post abortion	x		x		x	
	-Offer/make appointments for IUD-insertion post abortion directly at the time of abortion counseling	x		x		x	
	-Offer (“fast track”) insertion of IUD- or subdermal implants within a week post abortion.		N	x		x	
	-Increase skills training for midwives in LARC-insertion post abortion.	x		x		x	
	-Offer women appointments with midwives instead of physicians to achieve more timely IUD insertion					x	
**Improve counselling and services to women with communication needs**	-Schedule women who need an interpreter to appointments with midwives	x					
	-Refer Arabic-speaking women to written information and refer women to websites in different languages	x		x		x	

“New Test” (N) means that the team tested the change the time after the QIC. “Maintain” means that the team reported at 12 months follow up meeting, that they continue the change in practice. Categories and subcategories represent different steps the qualitative data analysis

### Improved patient outcomes and system performance regarding access to LARC

**Fig. 1:**
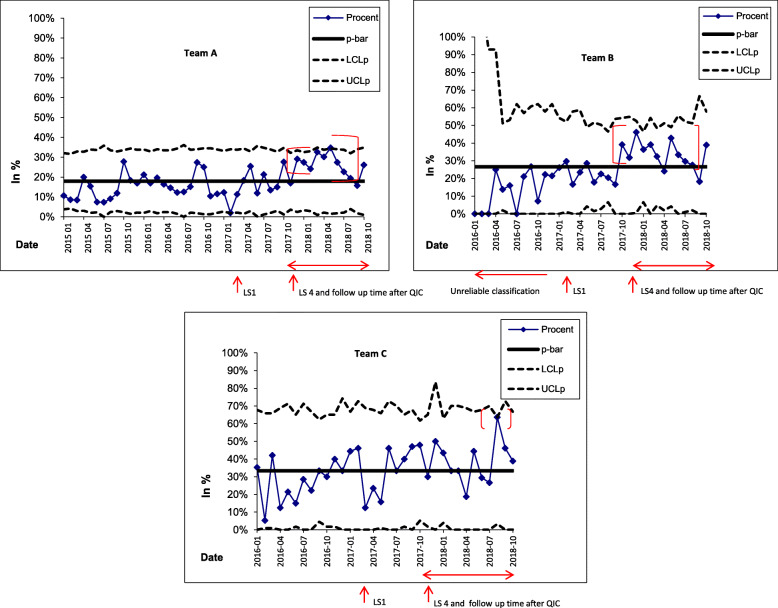
P-charts for teams A, B and C showing the monthly proportion (%, on the y-axis) of women who initiated LARC within 30 days post-abortion. For team B, the data recording was of uncertain reliability before the QIC. Data for teams A and B was extracted from existing electronic health information systems. For team C, data was manually collected from records prior to the QIC. Note the signs of special cause variation, highlighted in red, signalling an improvement in the proportion of women starting LARC in a timely manner. LARC = Long-acting reversible contraception. QIC = Quality Improvement Collaboratives. LS = Learning sessions. p-bar = the average of all observations. LCLp = Lower control limit (for proportions). UCLp = Upper control limit (for proportions)

**Fig. 2:**
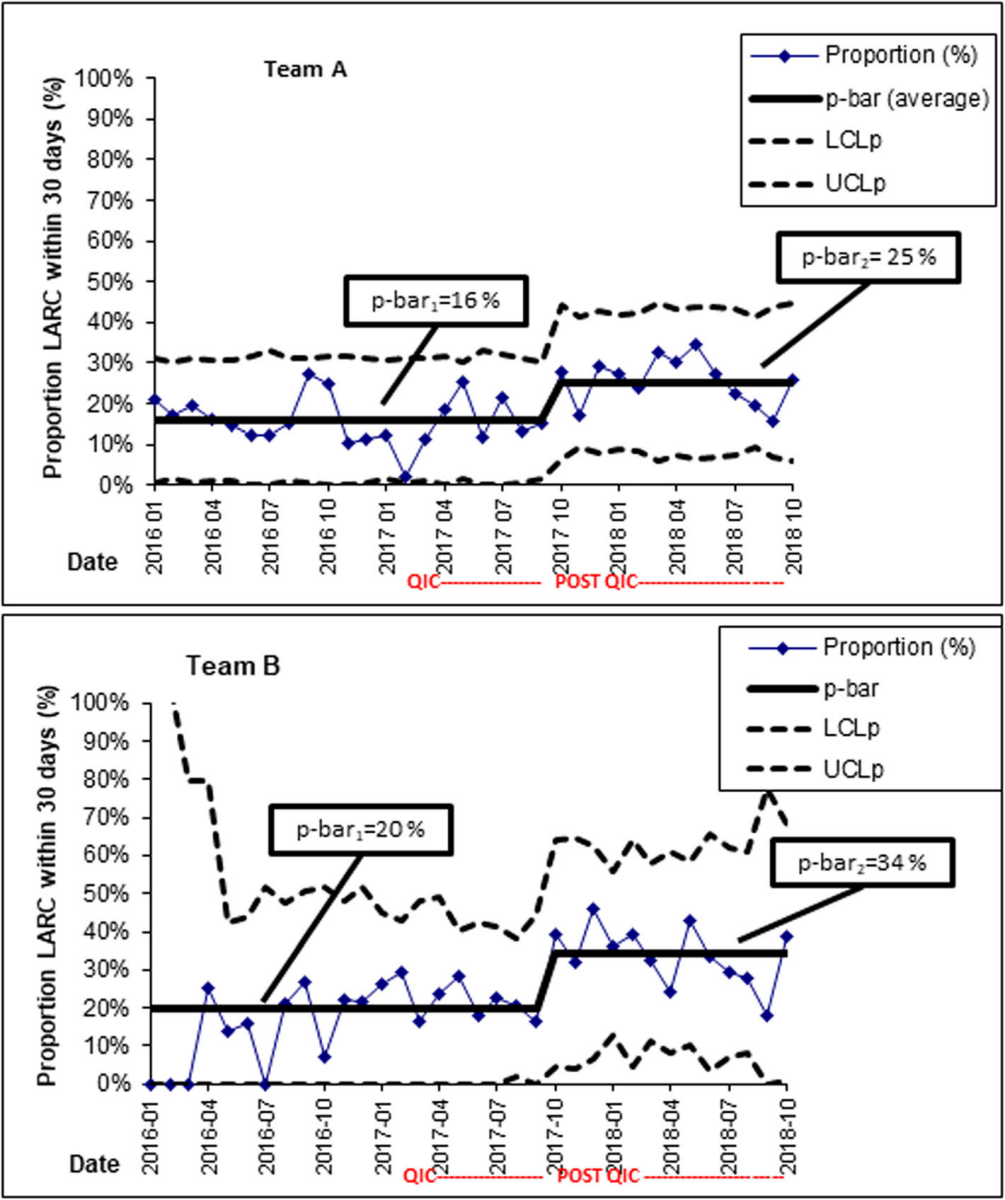
P-charts for teams A and B showing the monthly proportion (%, on the y-axis) of women who initiated LARC within 30 days post-abortion, with a re-calculated average for the Post-QIC period. This indicates the shifts (i.e. improvement) in the proportions of women who started use of LARC after the conclusion of the QIC, compared to before and during the QIC, for these teams. LARC = Long-acting reversible contraception**.** QIC = Quality Improvement Collaboratives**.** POST-QIC = The follow-up period 12 months after the conclusion of the QIC. LS = Learning sessions**.** p-bar = the average of all observations**.** LCLp = Lower control limit (for proportions)**.** UCLp = Upper control limit (for proportions)

The control charts for teams A and B signalled significant improvements compared to before and during the QIC, in the proportion of women who could initiate LARC within 30 days post-abortion, even if teams A and B did not achieve the 50% goal post-QIC (Figs. 1 and 2). This improvement was evident as a shift (upwards) in the proportions of women initiating LARC in the time after the QIC (Fig. 2).

Team C achieved the goal of more than 50% of women receiving LARC within 30 days post-abortion in a couple of separate months post-QIC, with large variations between individual months (Fig. 1) and not a consistent pattern of performing above 50%. In Fig. 1, the observation from August 2018 indicates a special cause as that observation is above the upper control limit. The underlying cause of this better-than-usual performance is not known.

This improvement in women’s timely access to LARC after the QIC (Figs. 1 and 2) was corroborated by HCPs’ reports that after the QIC, they had started to offer women appointments to initiate LARC within 10 days post-abortion (Table 1).

All teams reported that they had continued to improve contraceptive and abortion services after the QIC, notwithstanding high midwife turnover in two teams’ departments. Teams A and C reported improved collaboration with the Adolescent Health Services and midwifery clinics in their communities to provide access to LARC post-abortion. These services now also offered and supported fast track for LARC insertion within 10 days post abortion.

The teams also reported experiencing better teamwork and improved support from their department leadership.

*“We support each other much more and ask for help in difficult consultations to a greater extent now compared to before the QIC”*. A midwife in team X.

One team described how they could add more appointments for LARC insertion if they needed to; a new capability developed after the QIC.

*“Our clinical manager is now interested in the access to LARC post abortion, supports offering women appointments for insertion LARC post abortion and training of staff in LARC insertion. This changed after the QIC.”*A gynaecologist in team X.

### Professional development: experiences of professional development and improved system performance after the QIC

HCPs reported that they had continued the use of several approaches to contraceptive counselling developed during the QIC (Table 1).

*“We experience that women are better prepared for a conversation regarding contraception now.”* A midwife in team X.

They continued to focus on how to create trustful relationships when counselling women. HCPs also noted their needs for continuous training regarding how to develop trustful relationships and perform respectful counselling. They reported remaining challenges in counselling women who were sceptical towards using hormonal contraception after an unintended pregnancy. Furthermore, they reported that they reflected more on how to avoid pushing sceptical women to choose LARC against their will.

*“It is difficult to teach other HCPs how to establish trustful relationships when counselling women who are sceptical of contraceptive methods in the context of abortion.”* A midwife in team X.

Participating HCPs reported that they continued to practice how to insert LARC after the QIC. This training, which took time (several months) to organize, was fundamental for developing confidence in providing LARC.

They also reported a sense of meaning in providing and improving contraceptive services. They concluded that they were playing an important role now and that this was a new experience since the QIC.

## Discussion

In the 12 months after the QIC, all teams improved access to LARC and thereby increased the proportion of women who initiated LARC within 30 days post-abortion. This finding adds encouraging knowledge on how a QIC can enable better patient outcomes, system performance, and professional development, particularly in contraceptive services around the time of abortion. Our results are important, since access to LARC is crucial for improving women’s ability to prevent unintended pregnancies [3, 15]. We know that 45–50% of women who undergo an abortion return for subsequent abortions [5, 6, 24], that LARC is associated with a lower risk of unwanted pregnancy [10, 19, 25], and that both women and HCPs report barriers to LARC in the context of abortion [1, 7, 20, 26]. Therefore, improved counselling coupled with better access to LARC post-abortion should reduce the risk of unintended pregnancies, and thus promote women’s sexual and reproductive health.

Our study demonstrates that change for improvement can take time to materialise [12]. The teams and their departments needed more than the QIC’s 8 months duration to see the desired effect of several changes. This included the time to receive support from leadership to make changes in clinical practice [27, 28] regarding access to LARC [26]. In our study, support from leadership, including time for training, was fundamental to enable fast track access for LARC insertion within 10 days post abortion. Encouragingly, we found that most of the teams’ changes regarding contraceptive services achieved during the QIC were sustained 12 months after the QIC, despite a high midwife turnover in two teams. In that time, teams also introduced additional changes highlighted in the QIC, which they had not managed to introduce during it.

The participating HCPs also reported experiences of professional development due to participating in the QIC. The practical training in LARC methods was pivotal for them to gain confidence in providing LARC, mirroring findings from interviews in 2009 with clinicians in 25 abortion care practices across the USA [26]. Limited knowledge, practical skills training and confidence in providing LARC in abortion care may explain why some HCPs hesitate to recommend LARC or why some women do not receive LARC in a timely fashion post-abortion [26]. Furthermore, like Purcell et al. [9], we found that the HCPs saw a need for training in how to counsel sceptical women, also after the conclusion of the QIC. These findings imply that the HCPs in the abortion care have a continuous need for practical skills training in providing both respectful counselling and LARC.

### Methodological considerations and future research

A strength of this study is its longitudinal character, using quantitative data for the monthly proportion of women initiating LARC in a timely manner over more than 3 years. It is (hypothetically) possible that some of the women received LARC within 30 days post-abortion elsewhere. However, the vast majority of all abortion care services and LARC insertions in the hospitals’ catchment area are provided in public health care services.

We were not able to distinguish which change ideas were most, or least, effective when improving access to LARC, a common challenge with these studies. In future research there is a potential to separate varying impact of coinciding changes, e.g. by using Design of Experiments methodology [29].

Furthermore, we were not able to evaluate women’s experiences of contraceptive services or the proportion of women who underwent repeat abortions during the study period, as we did in an earlier medical record review study [19] and an interview study [7]. In future studies it would be of value to study the impact of person centred counselling regarding women’s satisfaction with contraception and services.

The case study design enabled us to fruitfully combine quantitative and qualitative data. That the teams participated in analysing the quantitative data served as a form of participant validation of the analysis. The multiple forms of data in our study enabled triangulation, strengthening both the study’s construct and internal validity [30].

Our findings can guide stakeholders in using a similar QIC approach, with appropriate contextual adaptation [31], to improve contraceptive counselling and services in abortion care. We encourage further studies evaluating QICs, and the changes promoted by the Driver diagram in the present case, in abortion care in a range of contexts. Efforts to better support women who are sceptical and have trouble finding a satisfactory contraceptive method should be prioritised since such women are at particular risk of undesired outcomes.

## Conclusions

A QIC regarding contraceptive counselling and women’s access to LARC post-abortion helped clinical teams initiate changes to simultaneously improve patient outcomes, system performance and professional development. Some aspects of improvement, such as offering appointments for LARC initiation in a timely manner, can take longer than an eight-month QIC to fully materialise and yield the intended benefits.

## Data Availability

The dataset generated and/or analysed during the current study are not publicly available due to restrictions from the Ethics Review Board, but can be made available to qualified researchers upon request, after approval from the Ethics board. HK should be contacted if someone wants to request the data.

## Abbreviations

QIC: Quality Improvement Collaborative
LARC: Long Acting Reversible Contraception
UP: Unintended Pregnancy
HCPs: Health Care Professionals
